# Energetic and atomic structural analyses of the screw dislocation absorption at tilt grain boundaries in BCC-Fe

**DOI:** 10.1038/s41598-022-25066-9

**Published:** 2022-12-09

**Authors:** Chiharu Kura, Masato Wakeda, Kazushi Hayashi, Takahito Ohmura

**Affiliations:** 1 Applied Physics Research Laboratory, Kobe Steel, Ltd., 1-5-5 Takatsukadai, Nishi-ku, Kobe, 651-2271 Japan; 2 Research Center for Structural Materials, National Institute for Materials Science, 1-2-1 Sengen, Tsukuba, Ibaraki 305-0047 Japan

**Keywords:** Mechanical properties, Atomistic models

## Abstract

The dislocation–grain boundary (GB) interaction plays an important role in GB-related plasticity. Therefore, an atomistic investigation of the interaction provides a deeper understanding of the strength and fracture of polycrystalline metals. In this study, we investigated the absorption of a screw dislocation with a Burgers vector perpendicular to the GB normal and the corresponding symmetric tilt grain boundaries (STGBs) in BCC-Fe based on molecular static simulations focusing on the STGB-dislocation interaction energy and atomistic structural changes at GB. The STGB-screw dislocation interaction depends on the energetical stability of the STGB against the GB shift along the Burgers vector direction. When the interaction exhibited a large attractive interaction energy, the dislocation dissociation and the GB shift along the Burgers vector direction occurred simultaneously. The interaction energy reveals that the interaction depends on the energetical stability of the STGB in terms of the GB shift in addition to the geometrical descriptor of the GB type, such as the Σ value. The same behavior was also obtained in the reaction when the second dislocation was introduced. We also discuss the screw dislocation absorption and rearrangement of the GB atomistic structure in STGB from an energetic viewpoint.

## Introduction

Steel has been conventionally used in automobiles, buildings, roads, and railways, among other infrastructures. There is significant demand for higher strength steel with a longer lifespan to help reduce its weight load and improve the safety of transportation equipment and social infrastructure. Because the properties of steel depend on its microstructure, selecting appropriate chemical compositions and manufacturing process conditions is critical. The microstructures of metals generally include various lattice defects, such as impurities, atomic vacancies, dislocations, and grain boundaries (GBs)^1^. Impurities in iron, such as sulfur and phosphorus, cause GB embrittlement^2,3^. In addition, hydrogen embrittlement is a significant problem in industrial applications in delayed fracture^4–6^. Because the suppression of brittle fracturing is an industrial issue, the factors influencing embrittlement, especially impurity concentrations and the decohesion mechanism, have been discussed^7–9^; in particular, the study of changes in interatomic bonds at the GBs based on the first-principles method has been reported^2^.

However, the effects of lattice defects on the fracture mechanisms of steel remain unclear. Even in brittle fractures at the GB, dislocation-based plastic deformation occurs near the GB before the final catastrophic fracture. The dislocation–GB interaction is an important factor in GB-related plasticity. Elucidating the dislocation–GB interaction is expected to provide a deeper understanding of the GB related strength and fracture of polycrystalline metals. For decades, various experimental and computational efforts have been made to unveil the dislocation–GB interaction^10–16^. From a practical perspective, dislocation–GB interaction is a key phenomenon in GB strengthening^17–19^. Therefore, atomistic dislocation analyses on far-field interaction with the GB, pileups near the GB, absorption at the GB, transmission across the GB, and nucleation from the GB have been conducted for crystalline metals. These previous works have primarily focused on face-centered cubic (FCC) metals, because the dislocation in an FCC metal has a wide core structure and is easily moved on an aimed slip plane under applied shear stress^20–27^.

In body-centered cubic (BCC) metals, screw dislocations dominate the mechanical behaviors. Screw dislocations have a remarkably low mobility and a cross slip mechanism can easily change the slip plane. Therefore, the atomistic evaluation of the screw-GB interaction under the applied shear stress is distinct from that of FCC metals. The dislocation–GB interaction in BCC metals requires further analysis to understand its fundamental nature.

In this study, we focused on the dislocation behaviors, especially for absorption and dissociation at the GB, and revealed the dislocation–GB interaction from the perspective of interaction energy in the system. Dislocation absorption is one of the basic phenomena of dislocation–GB interactions^28,29^. When a mobile dislocation approaches the GB, elastic interactions are generally induced. The elastic interaction often causes an energy barrier for dislocation absorption, as reported for FCC metals^30,31^. Therefore, elastic interaction is a key factor in dislocation absorption at the GB. In addition, the absorbed dislocation may induce changes in the atomic configurations at the GB, which affect the ability of GB for dislocation absorption and subsequent emission in the original grain or adjacent grain. Thus, the changes in atomic configurations at the GB due to the absorbed dislocations are significant factors. Dissociation of absorbed dislocation induces structural changes at the GB, and it has been reported in some atomistic studies^15,30^. Because dislocation dissociation affects strain accumulation and subsequent emission at the GB, an investigation of the dominant factors and background physics of the dislocation dissociation at the GB is important. To obtain atomistic knowledge on the absorption process of screw dislocation into the GB, we created screw dislocations and symmetric tilt grain boundaries (STGB) models and investigated the interaction between them using molecular static (MS) simulations based on the embedded atom method (EAM) potential. Since we here used large-scale atomic models, we chose the empirical force field model rather than more accurate ones such as the first principles calculation. We here intended to discuss the background physics of the relationship between the GB-dislocation interaction and the stability of GB. Herein, we focused on two factors: the elastic dislocation–GB interaction, and the structural changes caused by dislocation absorption. We used different types of GBs with different energetical stabilities, and evaluated the structural changes induced by the intense stress and strain fields of a screw dislocation. Moreover, we introduced a second dislocation into the model, in which the GB had already absorbed the first dislocation, to provide additional insight on the correlation between the GB structure and the structural changes at the GB caused by the screw dislocation. Finally, we discussed the fundamentals of screw dislocation absorption at the STGB based on the concept of the potential energy landscape.

## Results

### The interaction energies between the GB and the screw dislocation

**Figure 1:**
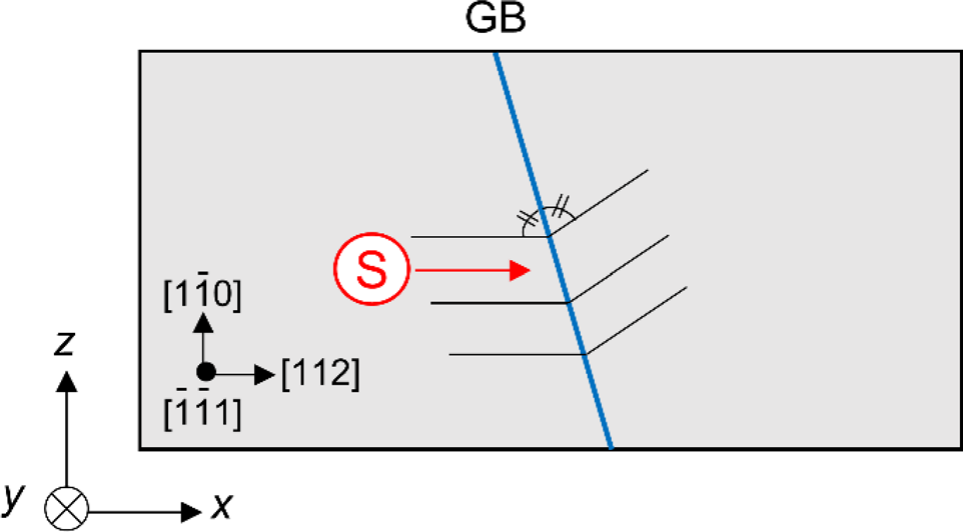
Schematic of the models for calculating GB-dislocation interaction. A letter “s” enclosed within a red circle shows position of the core of the screw dislocation.

**Figure 2:**
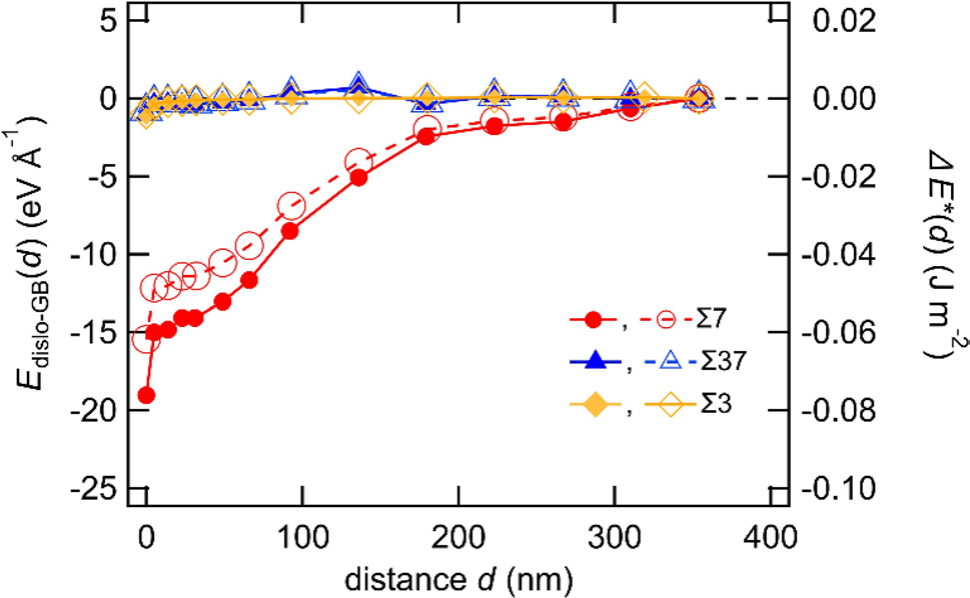
Changes in *E*_dislo-GB_(*d*) and ΔE*(*d*); *E*_dislo-GB_(*d*) is the interaction energy between a screw dislocation and GB. ΔE*(*d*) is the change in potential energy due to GB-dislocation interaction and has the same units as the GB energy. *E*_dislo-GB_(*d*) of Σ7 (filled red circle), Σ37 (filled blue triangle), and Σ3 (filled orange square) are plotted on the left axis, and ΔE*(*d*) of Σ7 (open red circle), Σ37 (open blue triangle) and Σ3 (open orange square) are plotted on the right axis.

In this study, we investigate the interaction between GBs and screw dislocations, which has a dislocation line direction along the <111> direction, using large-scale models to reduce the influence of the free boundary on the GB-dislocation interaction. Three <111> STGBs [Σ7(123)*θ* = 38.21°, Σ37(347)*θ* = 50.57°, Σ3(11$\overline{2}$)*θ* = 60.00°], of which properties have been investigated in computational studies^32–34^, were here chosen. We introduced the GBs in atomic models, as shown in Fig. 1. Figure 2 shows the interaction energy for Σ7, Σ37 and Σ3 GBs with the solid marks. The interaction energy for Σ7 GB decreases with decreasing *d*, and reaches a minimum value of − 19.0 eV Å^−1^ when the dislocation position is just above the GB, suggesting a large attractive interaction between Σ7 GB and the screw dislocation. The self-energy of the screw dislocation is approximately 1 eV Å^−1^ in this model system. Therefore, the significant decrease in the interaction energy for Σ7 GB implies that the GB structure becomes more stable by approaching screw dislocation. Meanwhile, in the case of Σ37 and Σ3 GBs, the interaction energy was almost constant regardless of *d*, indicating that the interaction energy of Σ37 and Σ3 GBs with the screw dislocation was small.

We also calculated ΔE*(*d*) = *E*_disl-GB_(*d*)/*S*, where *S* is the cross-sectional area of the GB in the GB model and ΔE*(*d*) is the dimension of the GB energy and reflects the change in potential energy due to the GB-dislocation interaction. In Fig. 2, ΔE*(*d*) with open marks shows energy profiles which are similar to the interaction energies. ΔE*(*d*) has a change in energy comparable to the calculated GB energies; the change in ΔE*(*d*) is much smaller than the GB energy even for Σ7 GB. Although the dislocation–GB interaction stabilizes the GB energy for Σ7 GB, as mentioned above, the energy change is insignificant compared to the GB energy due to the introduction of one dislocation in the large-scale GB model.

In previous studies on FCC metals, the elastic interaction between dislocations and GBs was repulsive, and an energy barrier was observed as the dislocations were absorbed at the GBs^30,31,35^. The repulsive interaction causes pile-up phenomena, which induces significant stress concentration and promotes dislocation transmission across the GB. In contrast, in the Σ7, Σ37 and Σ3 GBs of BCC-Fe considered herein, the dislocation–GB interactions do not show a significant energy barrier. In addition, the present work shows that the interaction differs depending on the GB types; the Σ7 GB shows a large attractive interaction energy, in contrast to that of Σ37 and Σ3 GB, wherein the interaction energy is negligibly small. Since Σ3 and Σ37 GBs exhibit similar energy profiles, we conducted detailed analyses for Σ37 GB.

### Stress field, sliding on GB plane, and local structure changes

**Figure 3:**
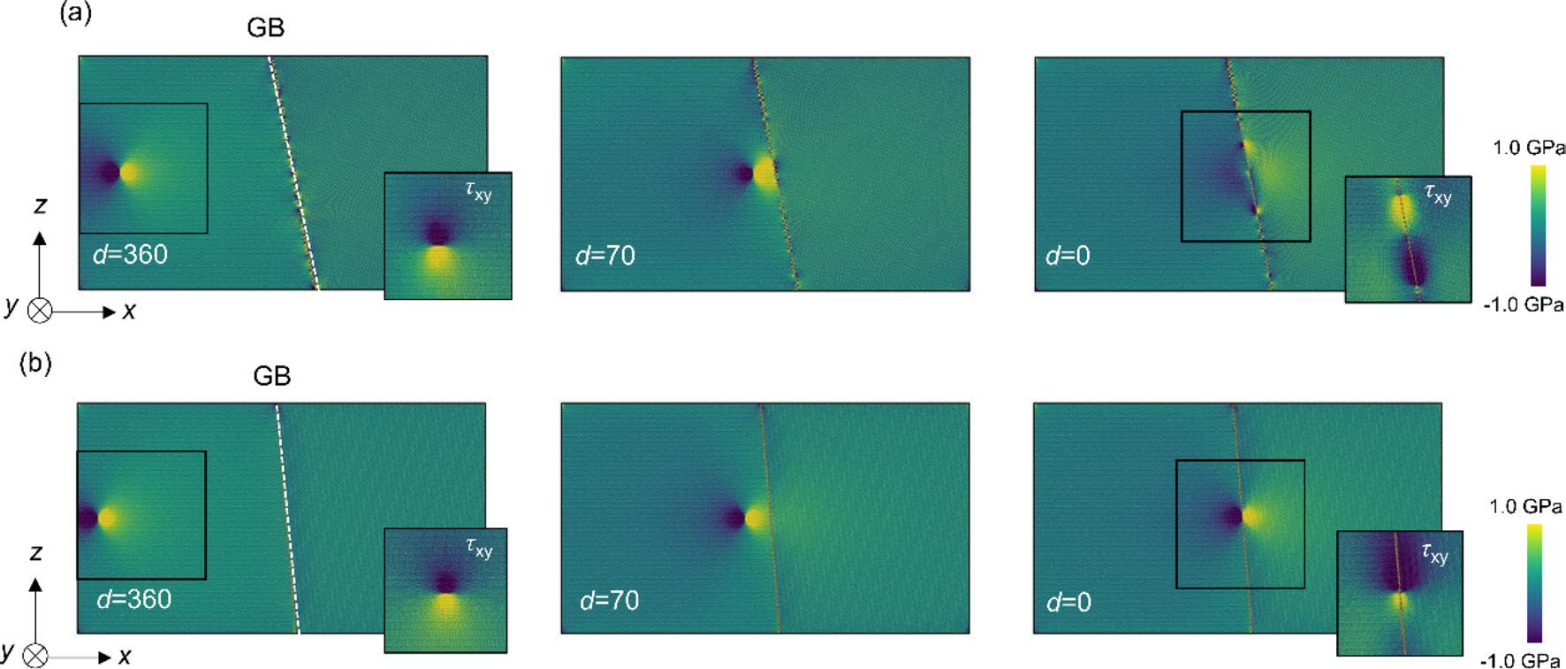
Changes in distribution of a stress component *τ*_*yz*_ as the screw dislocation approaches (**a**) Σ7 and (**b**) Σ37 GBs. The distance between the dislocation and GB, *d*, is depicted in each image. The embedded images at *d* = 0 and 360 nm show a distribution of the stress component *τ*_*xy*_. These images are simulated with LAMMPS (version 7Aug2019 https://lammps.sandia.gov/) and visualized using OVITO (version 3.1.1 https://www.ovito.org).

**Figure 4:**
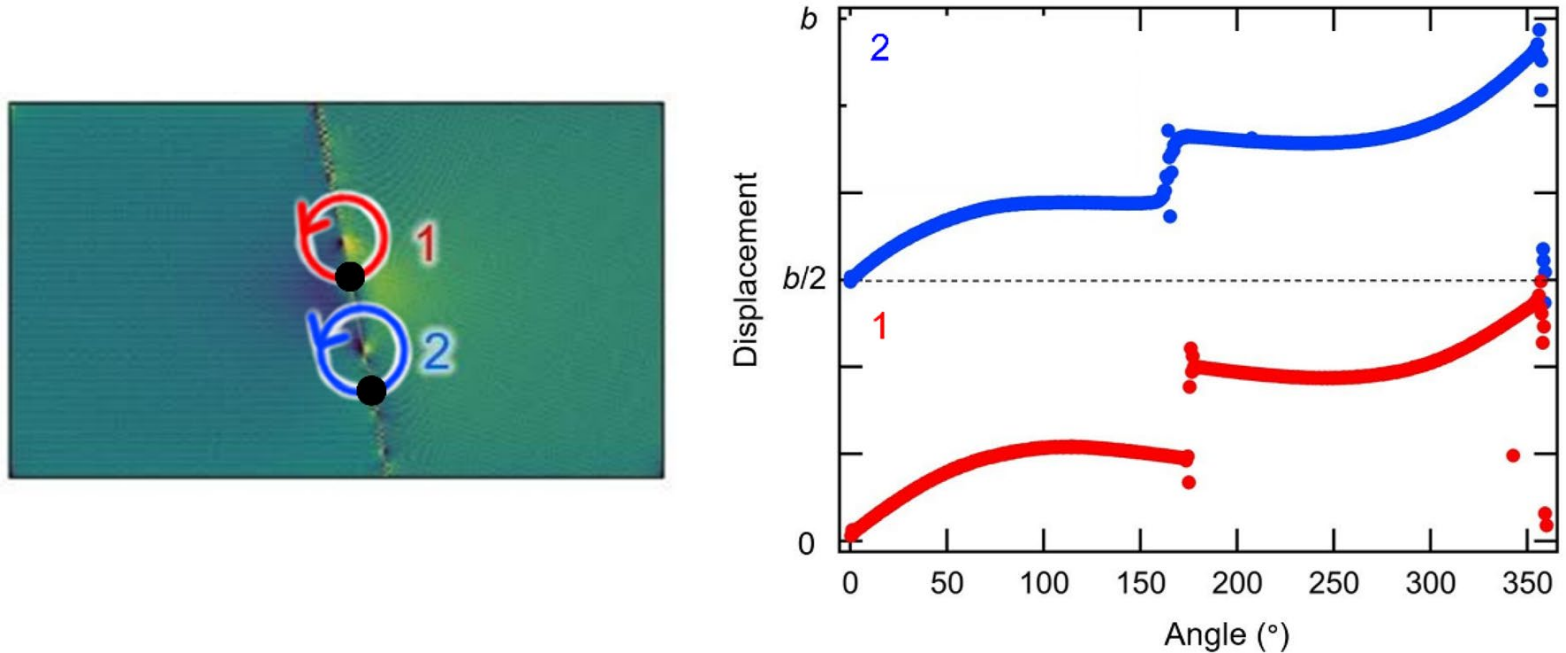
Burgers circuits around the two partial dislocations (1 and 2) in Σ7 GB model; The Burgers circuit around the partials was calculated counterclockwise, starting from the black dots of "1" (red) and "2" (blue) drawn on the embedded atomic figures. Each partial dislocation has *b*/2, and discontinuous displacement jumps were observed at around 180°, where it straddles the GB. The left image is simulated with LAMMPS (version 7Aug2019 https://lammps.sandia.gov/) and visualized using OVITO (version 3.1.1 https://www.ovito.org).

Stress distributions were also investigated in the dislocation–GB interaction analysis. The changes in the distributions of the stress component *τ*_*yz*_ are shown in Fig. 3 (additional images of Σ7 and Σ37 GBs are shown in Figure S1, and the stress distribution of Σ3 GB is shown in Figure S2 in Supplementary Information) wherein we used Ovito^36^ for visualization. In the case of Σ7 GB, no significant changes were observed in the stress distribution around the GB when *d* > 110 nm. The stress field of the screw dislocation then interacted with the GB when the distance became 70 nm or less. When the dislocation reached the GB (i.e., *d* = 0), a screw dislocation dissociated into two partial dislocations along the GB, and significant changes in the stress field at the GB were observed (Fig. 3a). Figure 4 shows the Burgers circuit around the two partial dislocations; there are *b*/2 helical displacements along the <111> directions revealing dislocation dissociation at the GB in case of the Σ7 GB. We in this study discuss the effect of local in-plane translation along the GB plane (i.e., GB shift) on the GB-dislocation interaction. In general, dislocation dissociation at GB is different from ones in perfect BCC structure due to the complex energy surface of GB shift. We also observed GB shift in directions other than *y*-direction, suggesting that the dislocation dissociation induces complex GB shift along the GB plane. Because the strain field of a dislocation changes considerably in Σ7 GB, the ease of displacement within the grain interface (i.e., GB shift) is important. In contrast, for the Σ37 GB, the stress field showed no significant changes, even when *d* = 0 (Fig. 3b). In addition, Σ3 GB also shows no significant changes, even when *d* = 0 (see in Figure S2 in Supplementary Information). These results show that the interaction between the screw dislocation and the GB differs depending on the GB type and agrees well with the results shown in Fig. 2.

**Figure 5:**
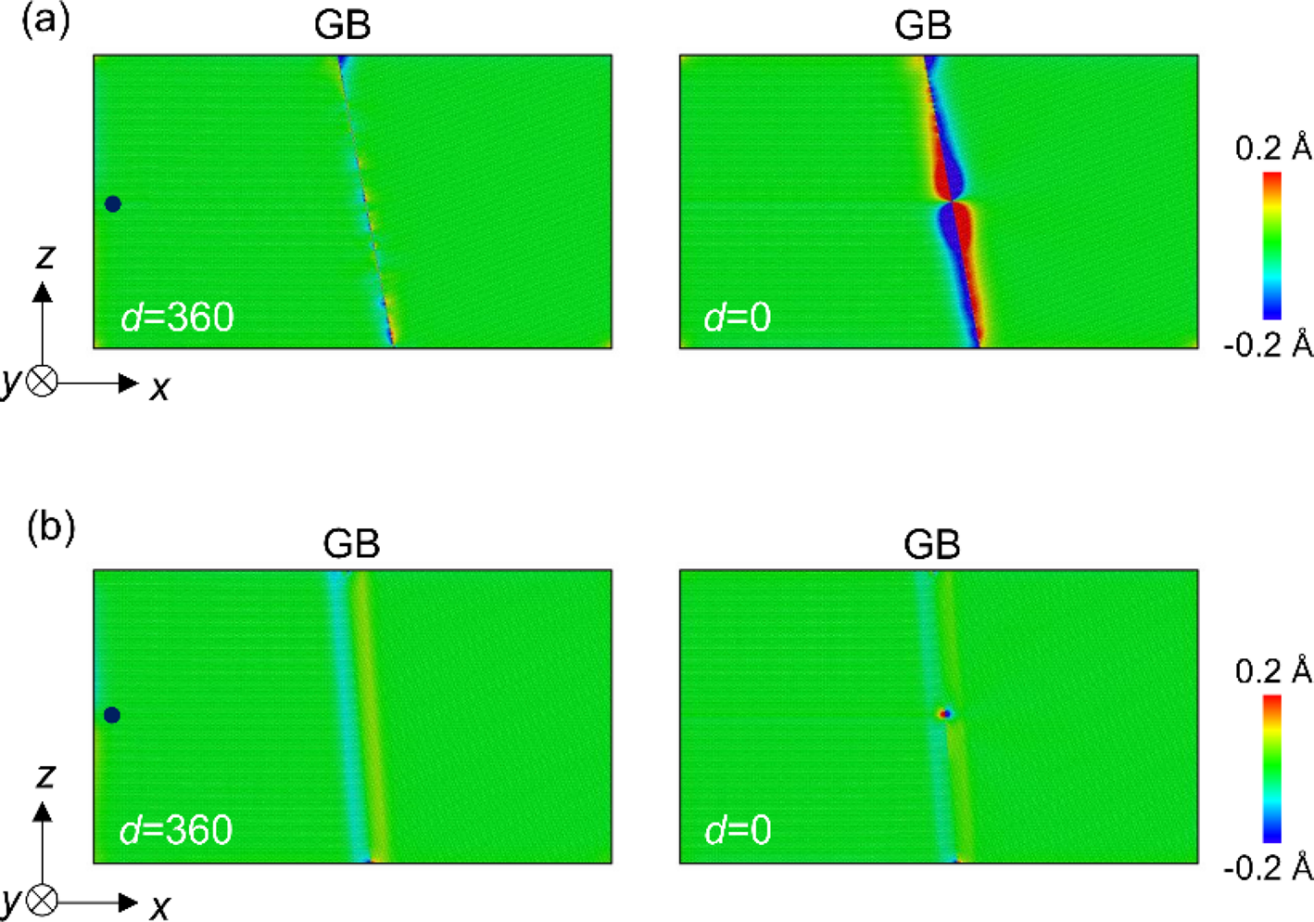
*δy*_*i*_ around (**a**) Σ7 and (**b**) Σ37 GBs. Black point shows a screw dislocation position of *d* = 360 nm. When *d* = 0, the screw dislocation is in the center of the GB in the *z* direction.

The changes in stress field shown in Fig. 3 indicate that the atomic structure of Σ7 GB changes by the screw dislocation absorption at GB. The displacement along the dislocation line direction (*y*-direction) was investigated using the atomic configuration of four models (1)–(4). (1) and (2) are models with a GB and no dislocations before and after relaxation, respectively; (3) and (4) are the models with the GB and dislocation at a distance *d* (nm) before and after relaxation, respectively. The *y*-coordination of atom *i* in model (*j*) is denoted by *y*_*i*_(*j*). Then, *δy*_*i*_= {*y*_*i*_ (4)-*y*_*i*_ (3)}-{*y*_*i*_ (2)-*y*_*i*_ (1)} represents the atomic displacement along the *y* direction induced by the effects of screw dislocation. The results for the Σ7 and Σ37 GBs are shown in Fig. 5. Regarding Σ7 GB, when the screw dislocation was introduced at *d* = 360, the atoms near the GBs shifted slightly along the *y* direction. Furthermore, when a screw dislocation was introduced just on the GB, the GB was helically shifted by approximately ± 0.6 Å along the *y* direction, of which the absolute shift between two grain (about 1.2 Å) consists of approximately half of the magnitude of the Burgers vector. The helical shift is an unusual GB shift and is caused by the screw dislocation, which has a helical strain field. The shifted region becomes smaller as it approaches the *z*-axis boundary of the model at Σ7 GB because the atomic shift was suppressed by the geometrical constraint at the boundaries. Similarly, in experiments, geometrical factors such as the triple point of GBs should affect the atomic shift. In the case of Σ37 GB, the GB is energetically stable before the introduction of dislocation. When a screw dislocation was introduced at distant positions or just on the GB, no significant change in *δy*_*i*_ was observed.

**Figure 6:**
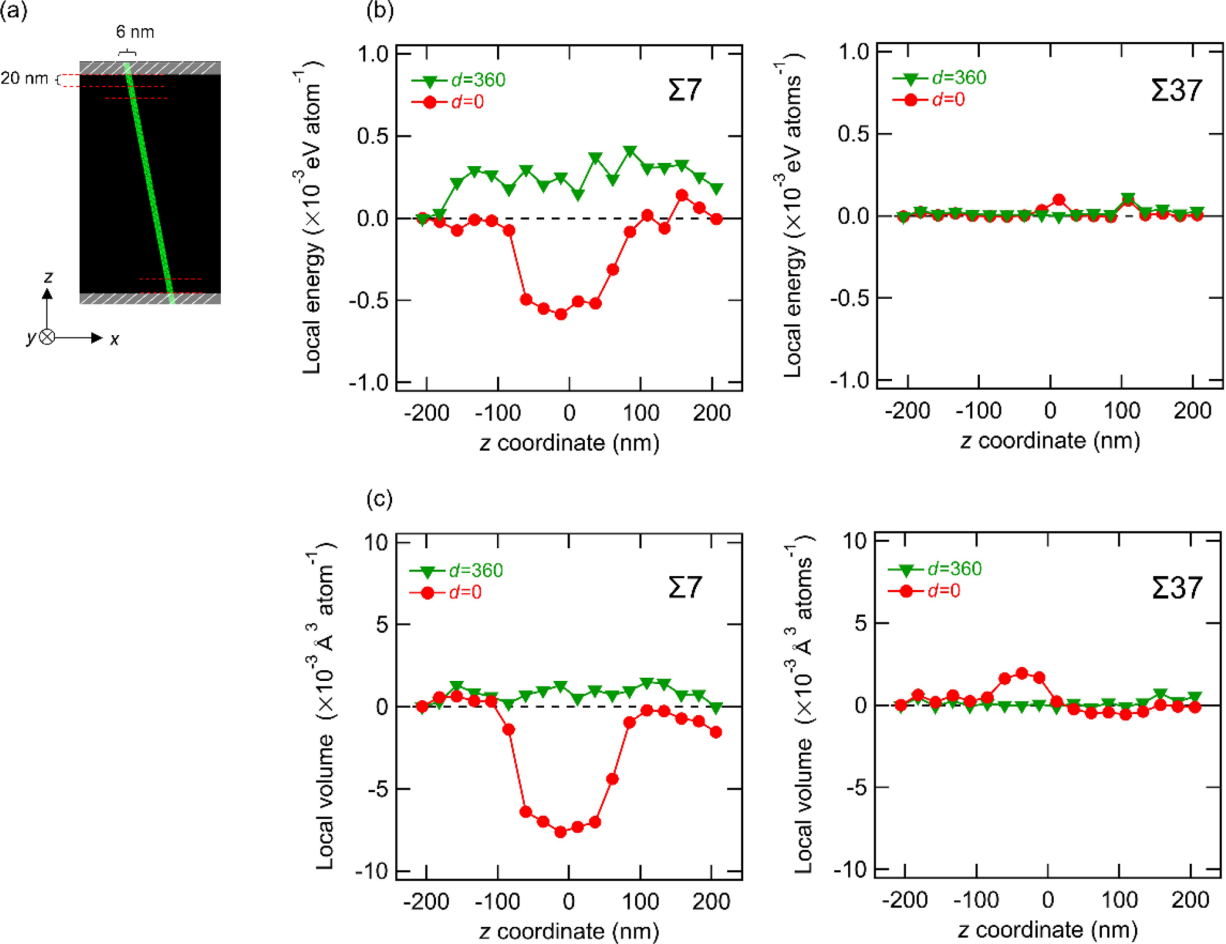
(**a**) Schematic of the local regions divided into 20 parts along the *z-*direction. White hatching areas are excluded due to the influence of the boundary condition. (**b**) Average atomic energy and (**c**) average atomic volume of each local regions near Σ7 and Σ37. The energy value farthest from the dislocation introduction point was set to zero.

For Σ7 and Σ37 GBs, local changes in the atomic potential energy and volume were investigated for *d* = 0 and 360 nm. As shown in Fig. 6a, approximately 360,000 Fe atoms located within 6 nm away from the GB along the *x*-direction were selected. The region near the *z*-axis model edges is affected by the boundary conditions, so the energy and volume calculation results near the *z*-axis boundary up to a distance of 20 nm were excluded.

The selected atoms were evenly divided into 20 regions based on their *z*-axis coordination, and the average atomic energy and volume in each region were calculated and are summarized in Fig. 6b, c. Regarding Σ7 GB, the local energy profile shows a significant decrement in the middle region along the *z*-axis when *d* = 0 nm (i.e., the dislocation is located just on the GB). Furthermore, the volume decreases in the same local regions when the dislocation is just on the GB. In other words, the screw dislocation just on the GB leads to an atomic configuration around the GB to a denser and more energetically stable state. The region with large changes in energy and volume is coincident with the region with a large *δy*_*i*_ in Fig. 5. Therefore, the significant changes in the local energy and volume are caused by the shift (or sliding) of the GB plane along the *y*-direction. Meanwhile, in the case of Σ37 GB, we cannot observe any significant changes in local energy and volume, agreeing well with the observations in Figs. 2, 3, and 5.

**Figure 7:**
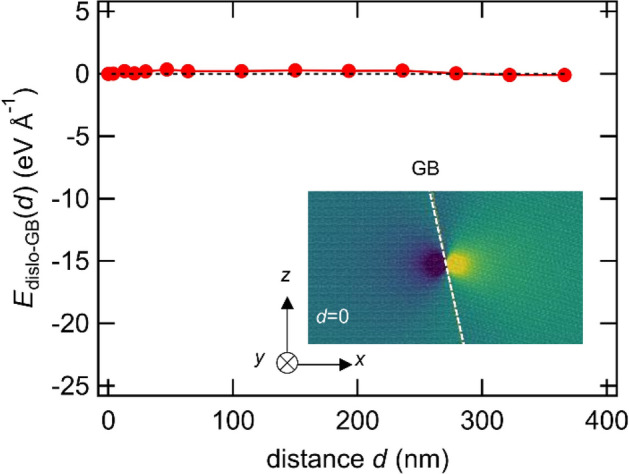
Interaction energy between stable Σ7 and the screw dislocation. The inset image shows a stress component when *d* = 0. The inset image is simulated with LAMMPS (version 7Aug2019 https://lammps.sandia.gov/) and visualized using OVITO (version 3.1.1 https://www.ovito.org).

The GB energy depends on the in-plane rigid body translation along the GB^37^. Even when the Σ value and crystallographic plane are determined, the GB may have various states in terms of the in-plane degree of freedom. To reveal the effect of initial in-plane rigid body translation along the GB, we prepared atomic models with different initial GB shifts along the *y*-direction (Burgers vector direction). As shown in Fig. 2, Σ7 GB is energetically “unstable” against the strain field by screw dislocation. As evaluated in the previous study, Σ7 GB is relatively more stable than other GBs with similar misorientation angles^33^. In contrast, in this study, the stability of GB is discussed based on resistance for GB shift along the GB plane induced by dislocation strain field rather than the relative GB energy. We have successfully constructed a “stable Σ7” model as below. One of the two grains of the unstable Σ7 GB was shifted by 0.8 Å in the *y*-axis direction before introducing dislocations. The GB energy of the stable Σ7 model was 0.04 J m^−2^ lower than that of the nominal configuration model (i.e., the Σ7 model used in Figs. 2, 3, 4, 5, 6 named “unstable Σ7”). The interaction energy between the stable Σ7 GB and the screw dislocation is shown in Fig. 7. The interaction energy was 0.09 eV Å^−1^, indicating that the interaction energy of stable Σ7 with the screw dislocation was small. Furthermore, the stress field in stable Σ7 showed no significant dislocation dissociation when it reached the GB (i.e., *d* = 0). The dislocation–grain boundary interaction in the stable Σ7 models is similar to that in the Σ37 model in Fig. 2. This result reveals that the additional degree of freedom (i.e., in-plane translation) has an important effect on the GB-screw dislocation interaction.

### The interaction between the GB and the second screw dislocation

As seen in the previous subsection, the GB-screw dislocation interaction depends on the in-plane translation at the GB as well as on the Σ value and crystallographic plane. Meanwhile, the dislocation absorption induces structural changes at the GB, which should affect the interaction between the GB and the next screw dislocation approaching the GB. The strain accumulation at the GB due to multi-dislocation absorption is an important factor for dislocation transmission across the GB. Hence, an understanding of the interaction between the GB and multi-dislocations is necessary.

**Figure 8:**
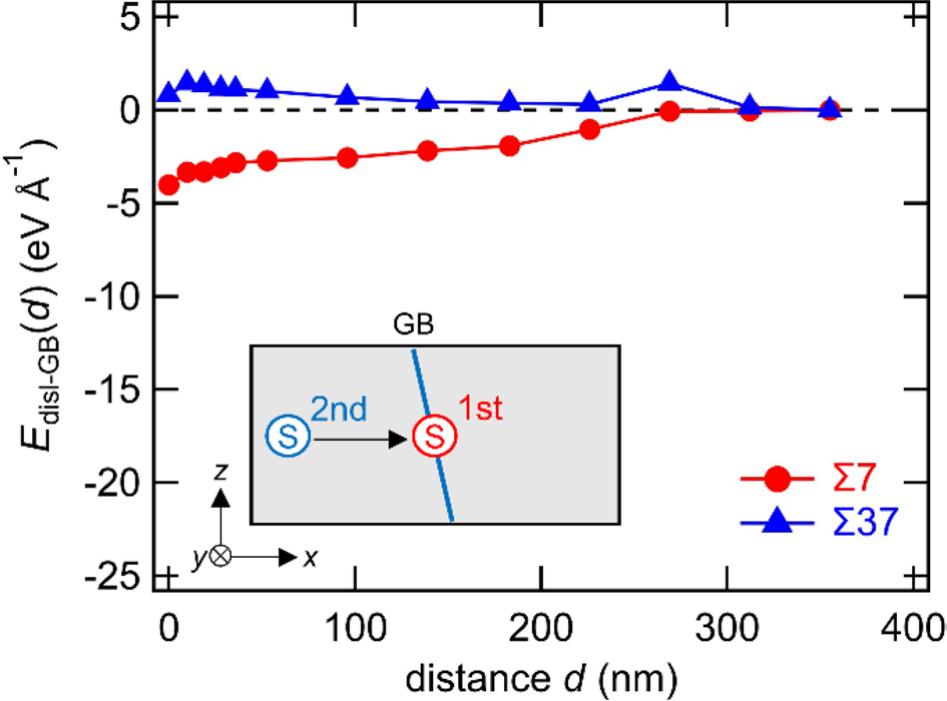
Interaction energy of second screw dislocation and GB. A letter “s” enclosed within a red circle shows the first screw dislocation, and a letter “s” enclosed within a blue circle shows the second screw dislocation.

**Figure 9:**
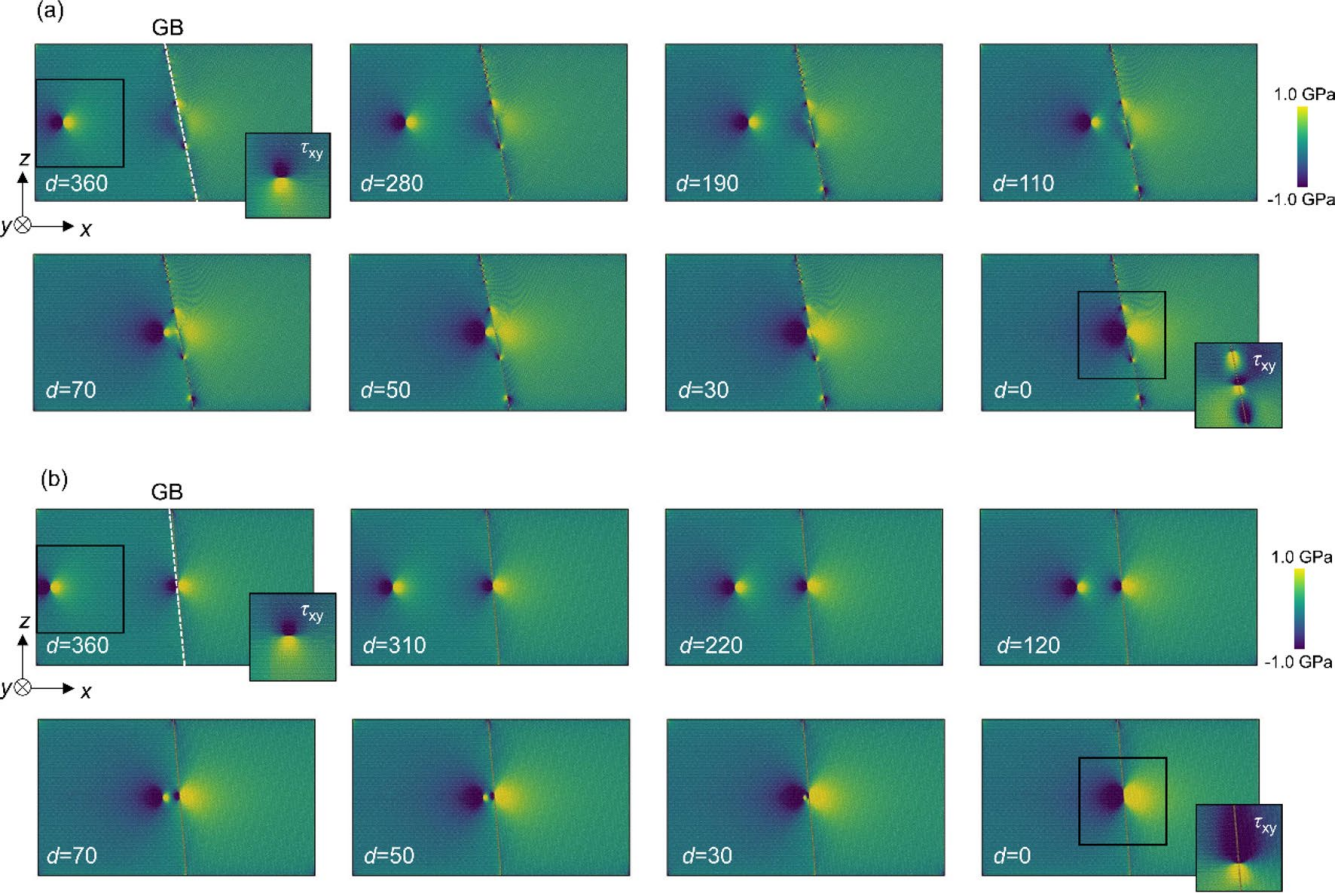
Distribution of a stress component *τ*_*yz*_ as the screw dislocation approaches to (**a**) Σ7 and (**b**) Σ37 GB in the model with the second screw dislocation. The distance between the dislocation and the GB are depicted in each image. The embedded images at *d* = 0 and 360 nm show the stress component *τ*_*xy*_. These images are simulated with LAMMPS (version 7Aug2019 https://lammps.sandia.gov/) and visualized using OVITO (version 3.1.1 https://www.ovito.org).

**Figure 10:**
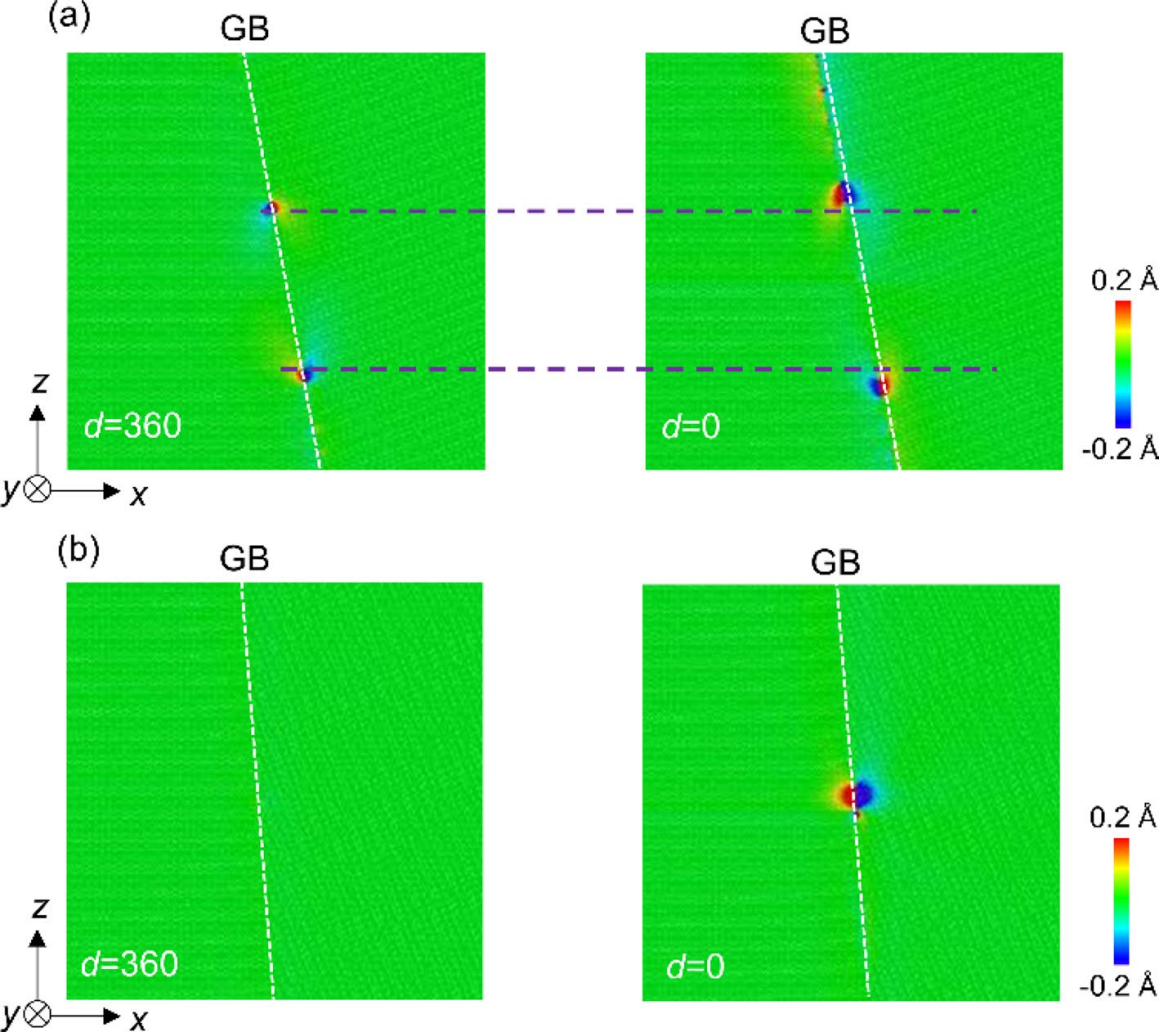
Distribution of *δy*_i_* in (**a**) Σ7 GB and (**b**) Σ37 GB models, which includes the second screw dislocations at *d* nm from the GB, as well as the first screw dislocation just on the GB. For the *δy*_i_* calculation, we used four models: (1) and (2) are the models which have a GB and the first screw dislocation absorbed at GB before and after relaxation, respectively. (3) and (4) are the models with the GB and the second dislocation at distance of *d* nm before and after relaxation, respectively. The *y*-coordination of the atom *i* in the model (*j*) are denoted by *y*_*i*_(*j*). *δy*_i_* = {*y*_*i*_ (4)-*y*_*i*_ (3)}-{*y*_*i*_ (2)-*y*_*i*_ (1)} represents the atomic displacement along the *y*-direction induced by the effects of the second screw dislocation.

**Figure 11:**
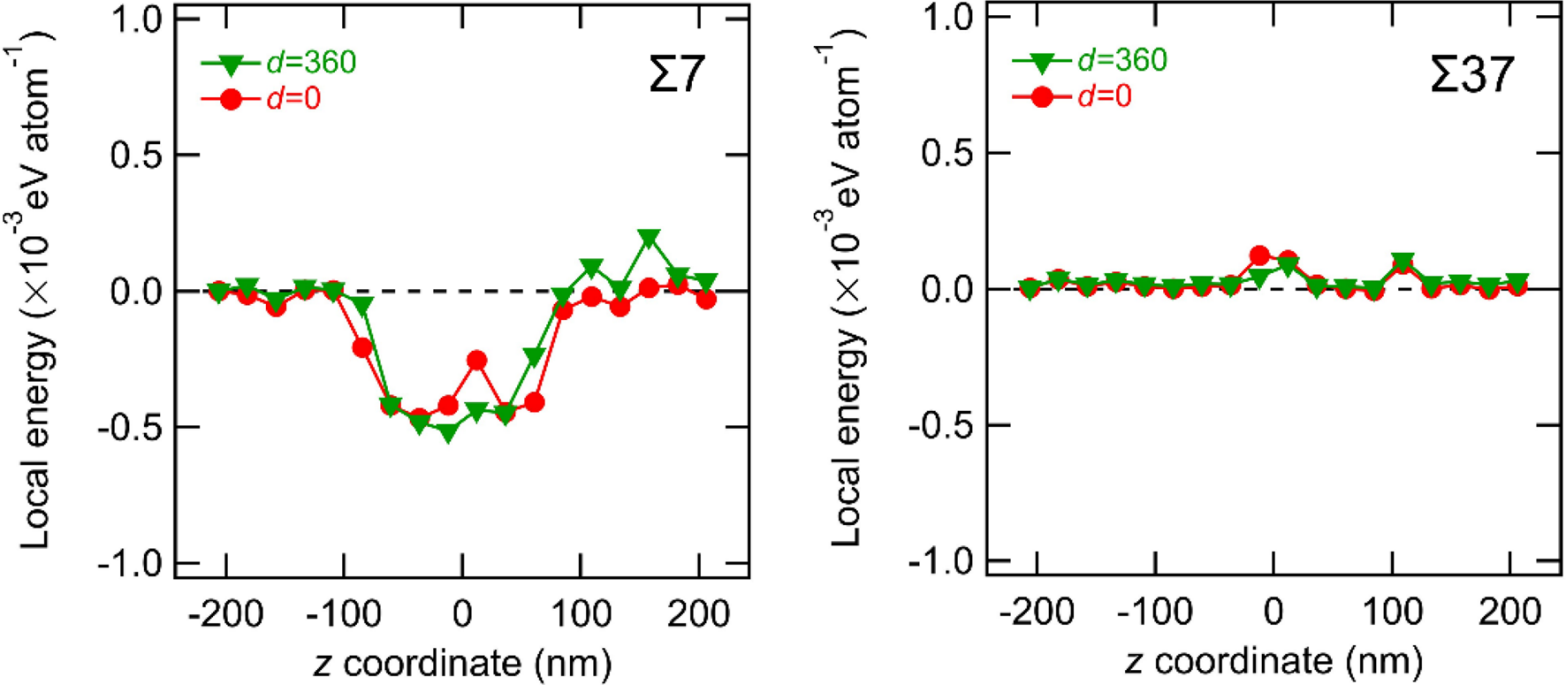
Change in average atomic energy of each local regions near Σ7 and Σ37 GBs induced by the second screw dislocations. The energy value farthest from the dislocation introduction point was set to zero.

For Σ7 and Σ37 GBs, we introduced a second screw dislocation in the model for which the first screw dislocation was already located just on the GB and the atomic configurations were fully relaxed. After introducing the second dislocation and sufficient relaxation at 0 K, we evaluated the interaction energy (Fig. 8), stress distribution (Fig. 9), atomic shift along the *y*-axis (Fig. 10), and average atomic energy (Fig. 11). In the case of Σ7 GB, there is an attractive interaction when the second dislocation approaches the GB, but the interaction energy is much smaller than that of the first dislocation (see Figs. 2, 8). In addition, we did not observe significant changes in stress distribution, atomic shift, or local energy, even if the second dislocation was located just on the GB. These results suggest that the second dislocation is not dissociated at the GB, and that it has a negligibly small effect on the stabilization of the GB. In the case of Σ7 GB, the GB is already stabilized by the GB shift owing to the stress (or strain) field caused by the first screw dislocation. Therefore, the stress (or strain) field caused by the second dislocation cannot induce a GB shift or the dissociation of a screw dislocation. In addition, when the second dislocation is at the *d* = 0 position, the region where the local energy decreases by the dislocation expands slightly. Therefore, the second screw dislocation decreases the potential energy of the model system when it is absorbed at the GB, as shown in Fig. 8. In the case of Σ37 GB, a marginal increase in the interaction energy was observed as the second screw dislocation moved toward the GB. Moreover, an atomic shift in the *y*-direction did not occur. The energy increase is caused by the elastic dislocation–dislocation interaction, which is a repulsive force. Therefore, when the first dislocation remains at the GB without dissociation, the second screw dislocation should be absorbed at a different GB site due to the repulsive dislocation–dislocation interaction and an easy cross-slip of the screw dislocation in BCC metals. The strain accumulation by absorbed dislocations is an important factor for dislocation transmission across the GB. For instance, it is possible that the strain accumulation by the absorbed multi dislocations at the same GB site enhances the dislocation emission in the adjacent grain. The present analyses of the second screw dislocation absorption at the GB imply that the accumulation is not easy in the case of screw dislocation due to the dissociation, repulsive interaction between screw dislocations, and cross-slip behavior of the inner grain. This suggests the dislocation type (i.e., dislocation component) affects the difference in both strain accumulation at the GB and dislocation transmission across the GB because the cross-slip frequency in BCC metals depends on the dislocation types. In BCC iron alloys, the difference in the dislocation transmission across the GB is indicated to depend on the GB types and dislocation types reacting with the GB^38^. The analysis of the second dislocation further suggests that the dislocation–GB interaction is dominated not only by the GB types (i.e., Σ value and crystallographic plane) but also by other factors correlated with the stability of the GB against the stress field of the dislocation.

## Discussion

In general, the GB is characterized by two parameters: “Σ value” and “crystallographic plane”. The former indicates the relative crystallographic orientation between the two grains. The latter indicates the crystallographic plane of the GB. Additionally, the GB has other degrees of freedom, such as in-plane rigid body translational positions between two grains, and local disorders at the GB. In this study, GB sliding (i.e., change in the in-plane translational position) along the *y*-direction was observed both in the initial relaxation and in the dislocation–GB interaction shown in Fig. 5. We found that the screw dislocation–GB interaction is affected by the translational degree of freedom along the *y*-direction, which is the direction of Burgers vector. In other words, the two ordinary parameters (Σ value and crystal plane) are not sufficient to explain the GB-dislocation interaction.

**Figure 12:**
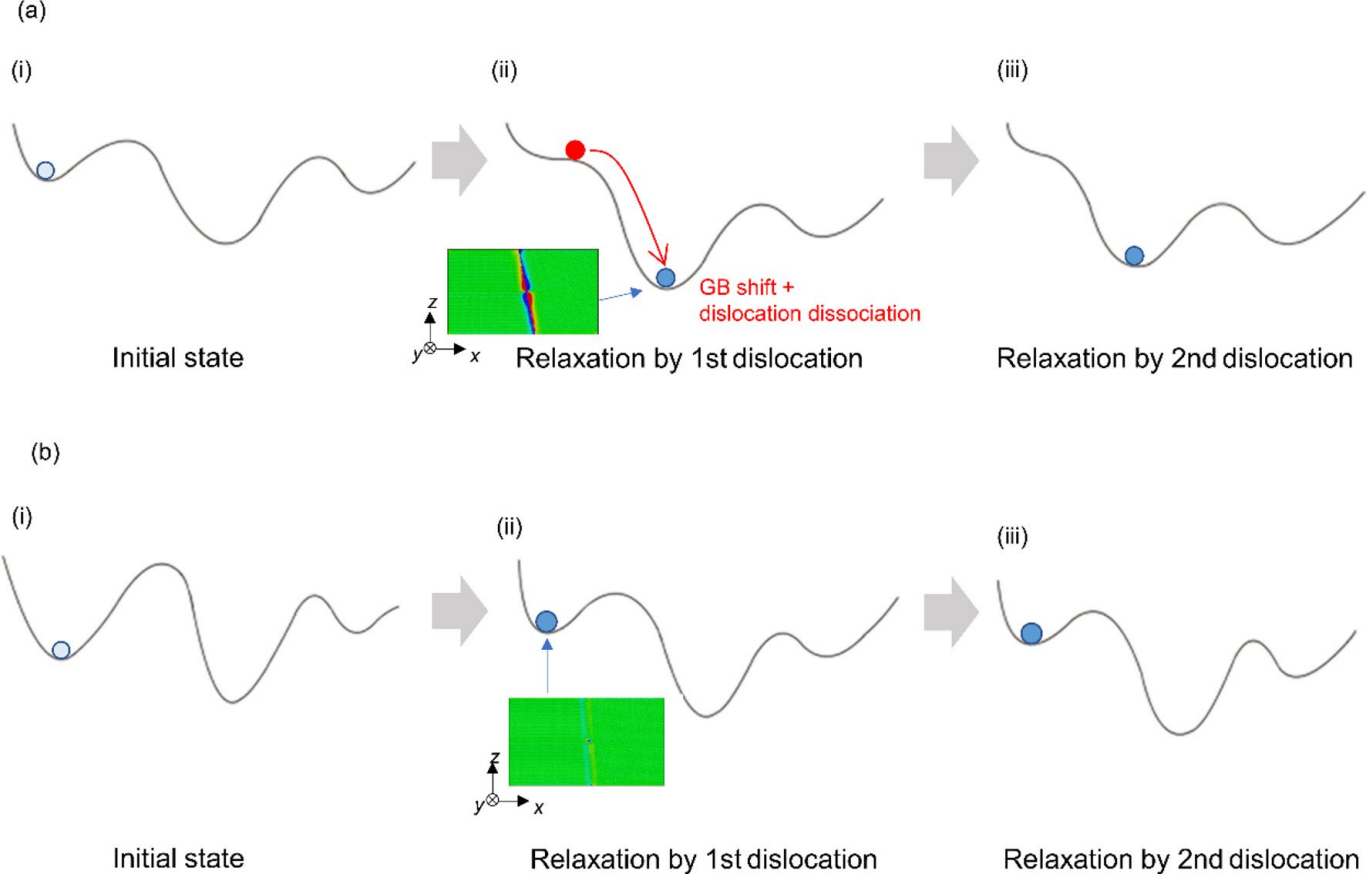
Schematic of the energy landscape for dislocation–GB system; (i) the initial state, (ii) relaxation by 1st dislocation, and (iii) relaxation by 2nd dislocation. (**a**) Energetically unstable GB and (**b**) energetically stable GB against screw dislocation absorption. The inset atomic models show GB shift and are the same as in Fig. 5; (**a**) unstable Σ7 and (**b**) stable Σ37.

An energy landscape perspective^39,40^ can provide general explanation on the dislocation–GB interaction based on the energetical viewpoint. Figure 12 schematically explains the initial state and dislocation–GB interactions for the two typical GBs based on the energy landscape. The shape of the energy landscape (e.g. basins and energy barriers) near the initial state, and the state with dislocation, dominate the dislocation–GB interaction. In the case of (a), it shows the initial energy state before introducing dislocation on small energy basins (i.e., state (i)). When a screw dislocation is introduced into state (i), the energy barrier for the transition to the nearest basin is reduced by the significant local shear stress of the screw dislocation, a GB shift along the *y*-direction and a dissociation of screw dislocation simultaneously occur (state (ii)), as shown in Figs. 4 and 5 (i.e., the unstable Σ7 GB case). In contrast, in the case of (b), similar to that in Σ37 GB and stable Σ7 (i.e., Σ7 mode used in Fig. 7), the initial state before introducing the dislocation is on the large energy basin (state (i)) When a screw dislocation is introduced into the GB models, the shear stress field caused by the screw dislocation cannot induce the GB shift and the dissociation of a screw dislocation does not occur (state (ii)). In this study, the second dislocation cannot induce a GB shift and dislocation dissociation, because the models are at large energy basin in the energy landscape in (a) and (b) (state (iii)).

This study suggests that the GB sliding and dislocation absorption depend on the in-plane rigid body translation, Σ value, and crystallographic plane. In addition, other lattice defects such as impurities and pre-absorbed dislocations affect the GB sliding and dislocation absorption at the GB. Different interatomic force fields also change dislocation–GB interaction and the shape of the energy landscape. Under the thermal environment, the local structural disorder at GB or GB sliding along the GB plane induced by thermal effects are additional factors. The analysis for such additional factors is important and would be future works. Thus, owing to the multiple factors involved, a clear understanding of the interaction has been challenging in the previous decades. We here demonstrated that the energetical stability of GB against the in-plane GB shift plays one of the key roles in understanding the dislocation dissociation. We suggest that the energy landscape based on the GB shift and dislocation dissociation can provide a phenomenological understanding of dislocation absorption at the GB.

## Conclusions

In this study, we investigated the interaction energy between screw dislocations and the STGB to understand the dislocation absorption behavior in BCC-Fe using MS simulations. The interaction can be evaluated using large-scale simulation models. The dislocation–GB interaction in BCC-Fe varies depending on the in-plane translation at the GB as well as the GB type denoted by the Σ value and crystallographic plane. In this model system, the unstable Σ7 GB (i.e., energetically unstable against GB shift) showed an attractive interaction with a screw dislocation, and the dislocation dissociated when it was absorbed by the GB. Moreover, a GB shift in the dislocation line direction (*y*-axis direction) was observed when screw dislocation was absorbed and dissociated at the GB. In contrast, the interaction energy of the stable Σ37 GB (i.e., energetically stable against GB shift) was negligibly small, and dislocations did not dissociate at the GB. When the second screw dislocation was introduced in the GB models, wherein the GB had already absorbed the first screw dislocation, no significant changes in stress distribution, atomic shift, and local energy were observed in both Σ7 and Σ37 GBs, even if the second dislocation was located just on the GB. These results suggest that the screw dislocation–GB interaction is dominated by the energetical stability of the GB structure against the local in-plane GB shift along the dislocation line direction in addition to the geometrical descriptor of the GB type, such as the Σ value.

## Materials and methods

The interaction energies between the corresponding symmetric tilt GBs with a common rotation axis of <111> and screw dislocation in α-Fe were evaluated by MS simulations. Three types of <111> STGBs [Σ7(123)*θ* = 38.21°, Σ37(347)*θ* = 50.57°, Σ3(11$\overline{2}$)*θ* = 60.00°], of which properties have been investigated in computational studies^32–34^, were used in this study. The GBs are represented by the “Σ value” and “crystallographic plane”. We introduced the GB structure in the models, as shown in Fig. 1. Note that the left grains of two models have the same crystallographic orientations: *x* $\left[ {112} \right]$, *y* $\left[ {\overline{1}\overline{1}1} \right]$, and *z* $\left[ {1\overline{1}0} \right]$. Meanwhile, the right grain has a different crystallographic orientation depending on the GB type.

The GB energy was calculated using relatively small bicrystal models consisting of two grains with approximately 20,000 atoms. The Σ7 GB energy was 1.14 J m^−2^, the Σ37 GB energy was 0.931 J m^−2^, and the Σ3 GB energy was 0.269 J m^−2^. The periodic boundary conditions were applied to all orthogonal directions, and the models were relaxed at 0 K under three-dimensional periodic boundary conditions.

The GB energy depends on the GB types denoted by Σ value and crystallographic plane^32,33,41,42^. In addition, the GB energy also depends on the in-plane rigid body translation along the GB^38^. The GB structures have an inherent in-plane degree of freedom in addition to Σ value and crystallographic plane. These structural degrees of freedom are determined during the microstructure formation process. Therefore, similar to Σ value and crystallographic plane, the in-plane degree of freedom may not always be energetically optimal during the microstructure formation process. In other words, even when Σ value and crystallographic plane are determined, the GB may have various states in terms of the in-plane degree of freedom. In conventional experimental and computational studies on the GB-dislocation interaction, the in-plane degree of freedom has not attracted much attention. Herein, in the cases of Σ7 and Σ3 GBs, no significant GB shift along the *y*-direction was observed during structural relaxation, without the effect of introducing screw dislocation. In contrast, in the case of Σ37 GB, a significant GB shift along the *y*-direction (± 0.4 Å) was observed during structural relaxation without the effect of introducing screw dislocation.

To analyze the interaction between a screw dislocation and GB, we used large bicrystal models with dimensions of 800 nm × 0.75 nm × 600 nm. The model consisted of approximately 26,000,000 atoms. Here, we used a small model dimension in the dislocation line direction and large model dimensions on the plane perpendicular to the dislocation line direction, which aims to maintain a large distance between the dislocation and model boundaries (model edges), thereby reducing the effect of the model boundaries on the evaluation of the interaction energy. A periodic boundary condition was applied only in the *y-*direction (i.e., the $\left[ {\overline{1}\overline{1}1} \right]$ direction). Meanwhile, in the *x* and *z* directions, specific boundary conditions were applied to the model edges; the positions of the edge atoms were fixed in the *x* and *z* directions, while positions in the *y* direction were free. To avoid atomic overlap around the GB, we excluded atoms which had neighboring atoms within 1.2 Å or less. Then, a screw dislocation, in which both the Burgers vector and line direction are parallel to the $\left[ {\overline{1}\overline{1}1} \right]$ *y*-axis, was introduced in the model. MS simulations were performed using the LAMMPS code^43^. The embedded atom method potential for BCC-Fe developed by Mendelev et al., which has been employed in the calculations of GB properties and GB-dislocation interactions, was used^44–46^. The quantitative results are expected to depend on the force field types (i.e., EAM potential, other types of interatomic potential, and first-principles calculation). Machine learning potentials have been recently developed for bcc iron, and they should be more accurate than conventional EAM potentials. Meanwhile, the calculation costs of the machine learning potentials are expensive for the present models, because we here used large-scale models to evaluate GB-dislocation interaction. In addition, we here intended to discuss the general physics of GB-dislocation interaction. The interaction energies between the GB and the screw dislocation (*E*_disl-GB_) were evaluated as a function of the dislocation–GB distance along the *x*-axis. We prepared several models in which the screw dislocation was introduced at a distance *d* (nm) from the GB (*d* = 0–360 nm; 0 nm means the screw dislocation is just on the GB). After introducing the dislocation, each model was relaxed using the conjugate gradient method at 0 K to obtain an energetically stable atomic configuration. The interaction energies of GB-dislocations (*E*_disl-GB_) were defined as follows:

1 $$E_{disl - GB} \left( d \right) \, = E\left( d \right) \, {-}E_{0}$$

where *E*(*d*) gives the potential energy of the models after relaxation, and *E*_0_ is the potential energy of the model with the largest *d* after relaxation. The interaction energies are defined as zero when the dislocation is located at the farthest position (i.e., ~ 360 nm). The dislocation–GB interaction is attractive when it has a negative value.

## Supplementary Information

Supplementary Information.

## Data Availability

The datasets generated and/or analysed during the current study are available from the corresponding author on reasonable request.
