# Supplementary material for: Energetic and atomic structural analyses of the screw dislocation absorption at tilt grain boundaries in BCC-Fe
Source: Sci Rep. 2022 Dec 9;12:21301. doi: 10.1038/s41598-022-25066-9 (PMC9734193; doi:10.1038/s41598-022-25066-9)
Supplement: Supplementary file 1 — Supplementary Information. [file 41598_2022_25066_MOESM1_ESM.docx]

*Supplementary Information*

Energetic and atomic structural analyses of the screw dislocation absorption at tilt grain boundaries in BCC-Fe

Chiharu Kura^1^, Masato Wakeda^2^, Kazushi Hayashi^1^, Takahito Ohmura^2^

^1^ Applied Physics Research Laboratory, Kobe Steel, Ltd., 1-5-5, Takatsukadai, Nishi-ku, Kobe, 651-2271, Japan

^2^ Research Center for Structural Materials, National Institute for Materials Science, 1-2-1, Sengen, Tsukuba, 305-0047, Japan

[**kura.chiharu@kobelco.com**](mailto:kura.chiharu@kobelco.com)**,** Applied Physics Research Laboratory, Kobe Steel, Ltd., 1-5-5 Takatsukadai, Nishi-ku, Kobe 651-2271, Japan.

[**WAKEDA.masato@nims.go.jp**](mailto:WAKEDA.masato@nims.go.jp), Research Center for Structural Materials, National Institute for Materials Science, 1-2-1 Sengen, Tsukuba, Ibaraki 305-0047, Japan.


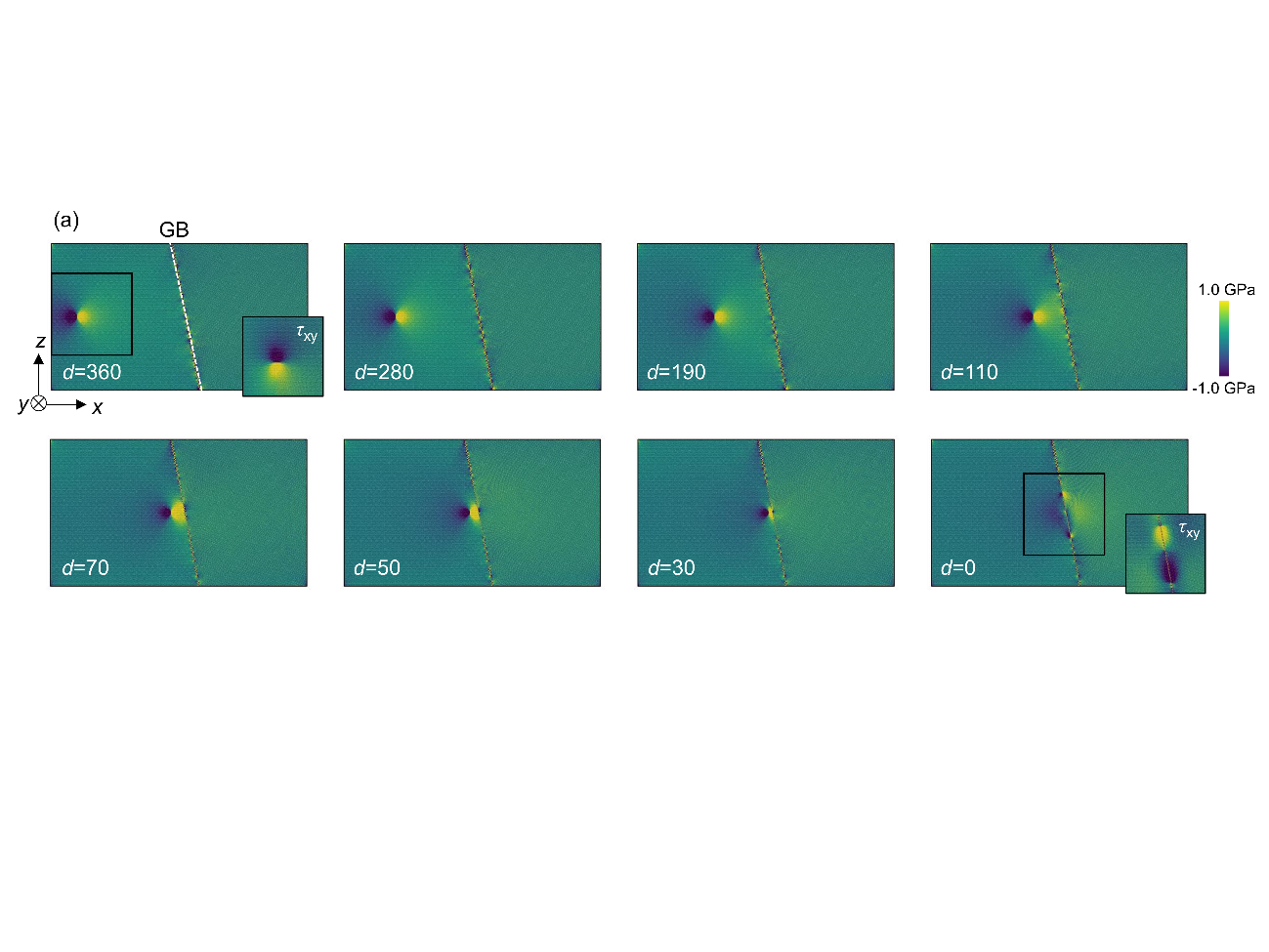


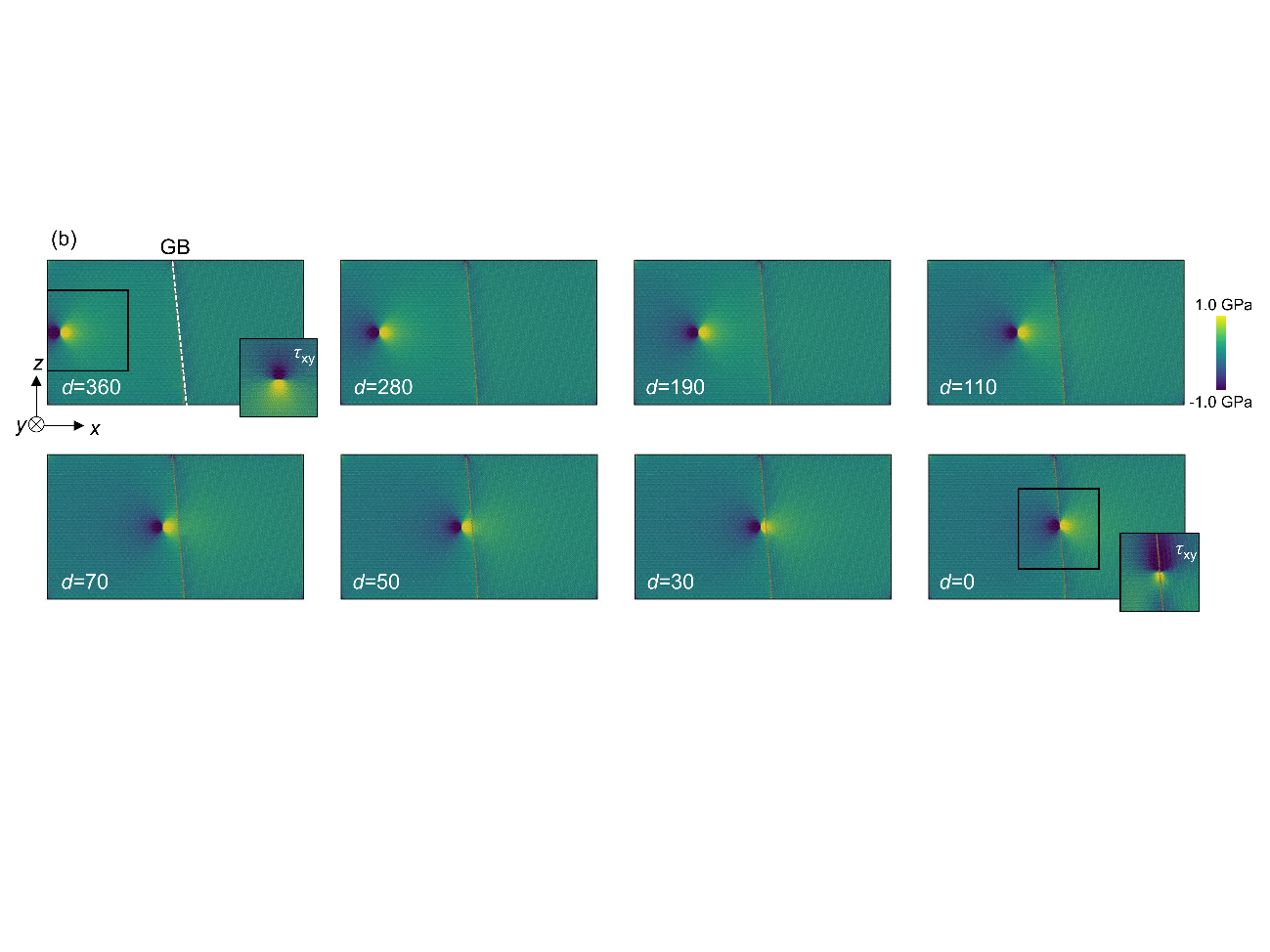


**Figure S1** Changes in the distribution of a stress component *τ_yz_* as the screw dislocation approaches (**a**) Σ7 and (**b**) Σ37 GBs. The distance between the dislocation and GB, *d*, is depicted in each image. The embedded images at *d* = 0 and 360 nm show a distribution of the stress component *τ_xy_*.


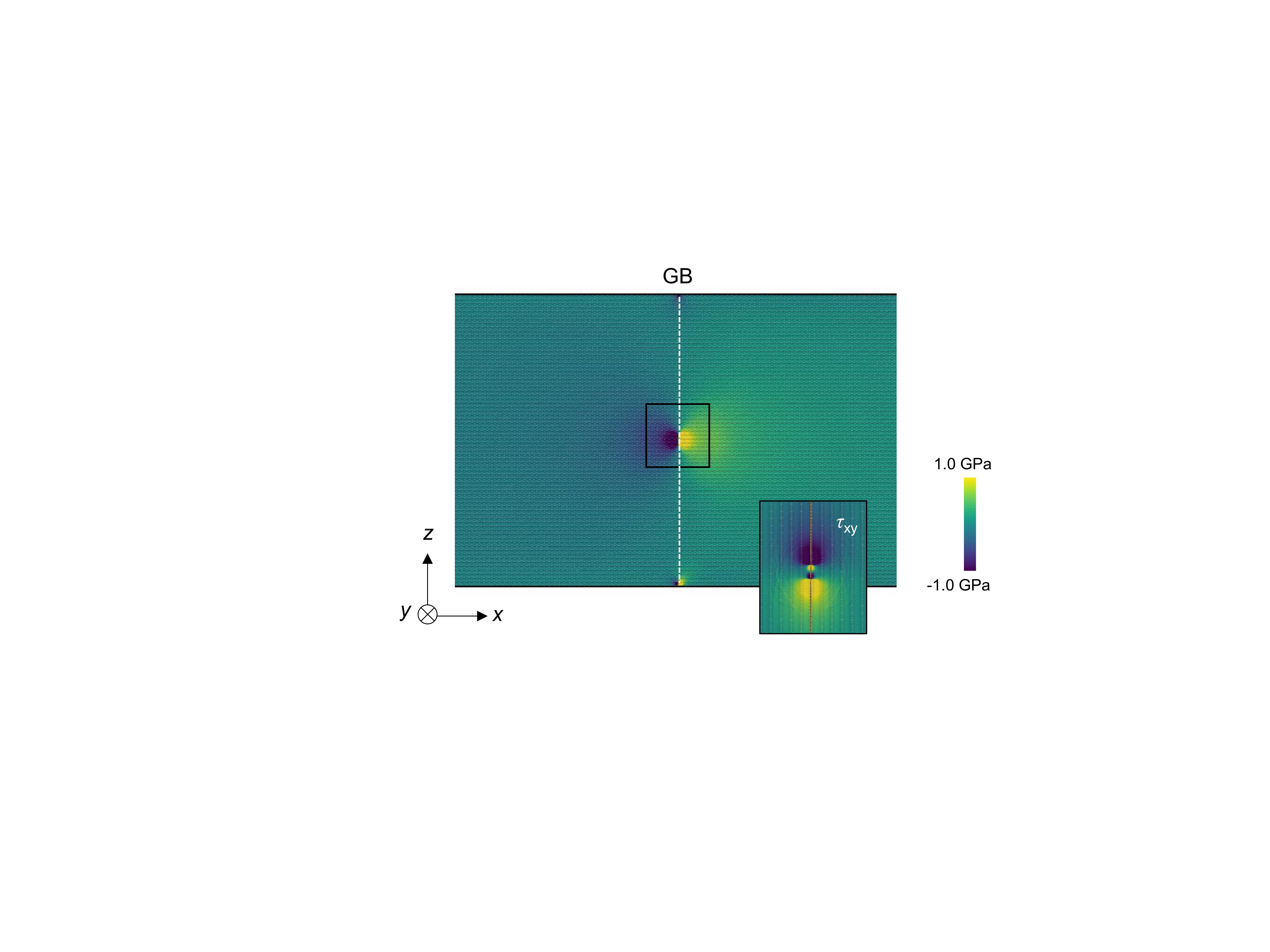


**Figure S2** The stress component *τ_yz_* as the screw dislocation locates just on Σ3 GB. The embedded image shows a distribution of the stress component *τ_xy_*.
